# Zirconia Nanoparticles as Reinforcing Agents for Contemporary Dental Luting Cements: Physicochemical Properties and Shear Bond Strength to Monolithic Zirconia

**DOI:** 10.3390/ijms24032067

**Published:** 2023-01-20

**Authors:** Anastasia Beketova, Emmanouil-Georgios C. Tzanakakis, Evangelia Vouvoudi, Konstantinos Anastasiadis, Athanasios E. Rigos, Panagiotis Pandoleon, Dimitrios Bikiaris, Ioannis G. Tzoutzas, Eleana Kontonasaki

**Affiliations:** 1 Department of Prosthodontics, School of Dentistry, Faculty of Health Sciences, Aristotle University of Thessaloniki, 54124 Thessaloniki, Greece; 2 Laboratory of Polymers Chemistry and Technology, Department of Chemistry, Faculty of Sciences, Aristotle University of Thessaloniki, 54124 Thessaloniki, Greece; 3 Department of Biomaterials, School of Dentistry, National Kapodistrian University of Athens, 2 Thivon Str., Goudi, 11527 Athens, Greece; 4 Department of Comprehensive Dentistry, College of Dentistry, Texas A&M University, 3000 Gaston Avenue, Dallas, TX 75226, USA; 5 Department of Operative Dentistry, School of Dentistry, National Kapodistrian University of Athens, 2 Thivon Str., Goudi, 11527 Athens, Greece

**Keywords:** zirconia nanoparticles, luting cements, bond strength, film thickness, flexural strength, thermocycling, water sorption, dual-curing, FTIR

## Abstract

Nanofillers in resin materials can improve their mechanical and physicochemical properties. The present work investigated the effects of zirconia nanoparticles (NPs) as fillers in commercial dental luting cements. Two dual-cured self-adhesive composites and one resin modified glass ionomer (RMGI) luting cement were employed. Film thickness (FT), flexural strength (FS), water sorption (*W_sp_*), and shear bond strength (SBS) to monolithic zirconia were evaluated according to ISO 16506:2017 and ISO 9917-2:2017, whereas polymerization progress was evaluated with FTIR. Photopolymerization resulted in double the values of *DC*%. The addition of 1% wt NPs does not significantly influence polymerization, however, greater amounts do not promote crosslinking. The sorption behavior and the mechanical performance of the composites were not affected, while the film thickness increased in all luting agents, within the acceptable limits. Thermocycling (TC) resulted in a deteriorating effect on all composites. The addition of NPs significantly improved the mechanical properties of the RMGI cement only, without negatively affecting the other cements. Adhesive primer increased the initial SBS significantly, however after TC, its application was only beneficial for RMGI. The MDP containing luting cement showed higher SBS compared to the RMGI and 4-META luting agents. Future commercial adhesives containing zirconia nanoparticles could provide cements with improved mechanical properties.

## 1. Introduction

Since the introduction of zirconia in restorative dentistry as an alternative to metal frameworks, the optimization of bond strength to this new material still remains a popular area of research that has expanded to all generations of zirconia materials including monolithic prosthetic restorations [1,2]. The zirconia in vitro bond strength testing methods vary and may influence the results [3]. Although many in vitro and in vivo experiments have been conducted and researchers have already introduced several different combinations of surface conditioning methods and luting materials, the results have not led to a method that has been universally accepted [4,5,6].

Translucent cubic zirconia materials and hybrid multilayered zirconia materials have recently been introduced to further zirconia indications and meet all posterior and anterior restoration demands. The adhesive potential of these new materials can be different due to their different chemical composition [7]. 

High strength ceramics, especially zirconia, have been characterized as inert materials. The increased fracture toughness, hardness, and absence of a glass phase result in a surface that requires high energy levels to be modified [8,9]. Increased surface roughness is very important to obtain micromechanical retention with any adhesive, however, the contribution of the chemical factor based on the composition of the primer, or the luting agent, is crucial [10,11,12]. The rheological properties, mechanical properties, and the chemical composition of the luting agent influence the adhesion potential to challenging zirconia surfaces [13,14].

Older generations of luting cements have been characterized as materials with poor mechanical properties and high sensitivity to water sorption [15,16]. Glass ionomer cements have demonstrated a significant increase in adhesion and minimal film thickness [17]. The development of resin-modified glass ionomers improved the mechanical properties of glass ionomers, but the first generation presented a high tendency to water sorption [18], which is now reduced in contemporary materials [15,19]. Moreover, contemporary resin cements, although more viscous than older generations, present extremely low film thickness in most commercial products due to the smaller filler size [20]. The optimum thickness for the clinical use of resin cements, depending on the adhesive substrate, is less than 100 μm [21].

The role of the luting agent is to embrace a dental prosthesis to a dental abutment, to prevent microleakage, and withstand chemical dissolution in the hostile oral environment [17]. Doubtful chemical affinity of the available luting cements and adhesive primers have urged the investigation to seek modifications in the chemical composition of many well-established commercial products to achieve a higher compatibility to zirconia substrates. Most manufacturers have followed this trend and modified their chemical composition where two basic trends have been adopted: either the incorporation of reactive adhesive monomers in the bulk of luting materials or accompanying these materials with specialized liquid primers that contain reactive monomers [6]. 

Notwithstanding, a new trend in research is the reinforcement of dental restorative materials with different inorganic fillers, apart from traditional amorphous glasses. The target is to increase the mechanical properties of these materials. Recently, nanoparticles have been integrated in glass ionomer resin cements to enhance their mechanical properties with promising results [22]. Moreover, zirconia nanoparticles have been used to strengthen adhesives [23], in resin restorative materials, and core build-up materials [24,25,26]. Moreover, favorable data have recently been published following the integration of nanoparticles in high impact heat-cured acrylic resin (PMMA) [27,28]. 

Until now, zirconia (ZrO_2_) particles stabilized with tetragonal yttria (Y_2_O_3_) have not been utilized for the reinforcement of a luting cement. However, zirconia-based nanoparticles are applied in restorative dentistry to improve the mechanical and antibacterial properties of different resin-based materials [29]. The most frequently reported methods for ceramic nanoparticles fabrication include sol–gel synthesis, co-precipitation, hydrothermal, spray-drying, spray pyrolysis, and freeze-drying. By sol–gel synthesis, uniform, nano-sized powders with high purity can be produced [30].

Particularly for the reinforcement of resin luting cements, ZrO_2_-based nanofillers could be beneficial for the establishment of durable bonds to zirconia-fixed restorations. Adhesive monomers containing phosphates, especially 10-MDP, create chemical bonds with metal and zirconia substrates [31,32]. There is strong evidence that 10-MDP creates both ionic and hydrogen bonds with zirconia. Moreover, the concentration and purity of 10-MDP is important since at least 1% wt. seems to be efficient in SBS tests [32]. On the other hand, as early as in 1978, Takeyama et al. added a carboxylic adhesive monomer (4-META) to increase the bond strength between enamel and acrylic resin [33]. Later, this monomer was incorporated in commercially available resin cements, and it is surprising that 4-META products have had the same ingredients since their inception [34]. It is well-established that carboxylic acids can bond to oxidizable metals such as aluminum oxide [35]. In a theoretical model, the insertion of zirconia nanoparticles could enhance the cohesive strength and alter the physicochemical properties of resin cements containing adhesive monomers. Increased mechanical properties in luting materials may promise bond strength durability after aging. The influence of inorganic fillers in any changes of the degree of conversion during the polymerization of dental composites is usually small. Nano-powders, specifically, do not hinder irradiation diffusion in the restoration and thus favorable curing and bond opening is achieved [36,37]. 

The aim of this research was the characterization of the physico-mechanical properties of zirconia nanoparticles reinforcing contemporary luting cements and the evaluation of the shear bond strength to translucent zirconia. The first null hypothesis of this study is that reinforcement with zirconia nanoparticles does not influence either the physicochemical properties or the polymerization progress of the luting agents tested. The second null hypothesis is that the shear bond strength of three different commercial luting cements to zirconia is not influenced by the integration of zirconia nanoparticles. The third null hypothesis is that the SBS of cements reinforced with zirconia nanoparticles is not influenced by thermal cycling.

## 2. Results

### 2.1. Fourier Transform Infrared Spectroscopy (FTIR) Characterization

**Figure 1:**
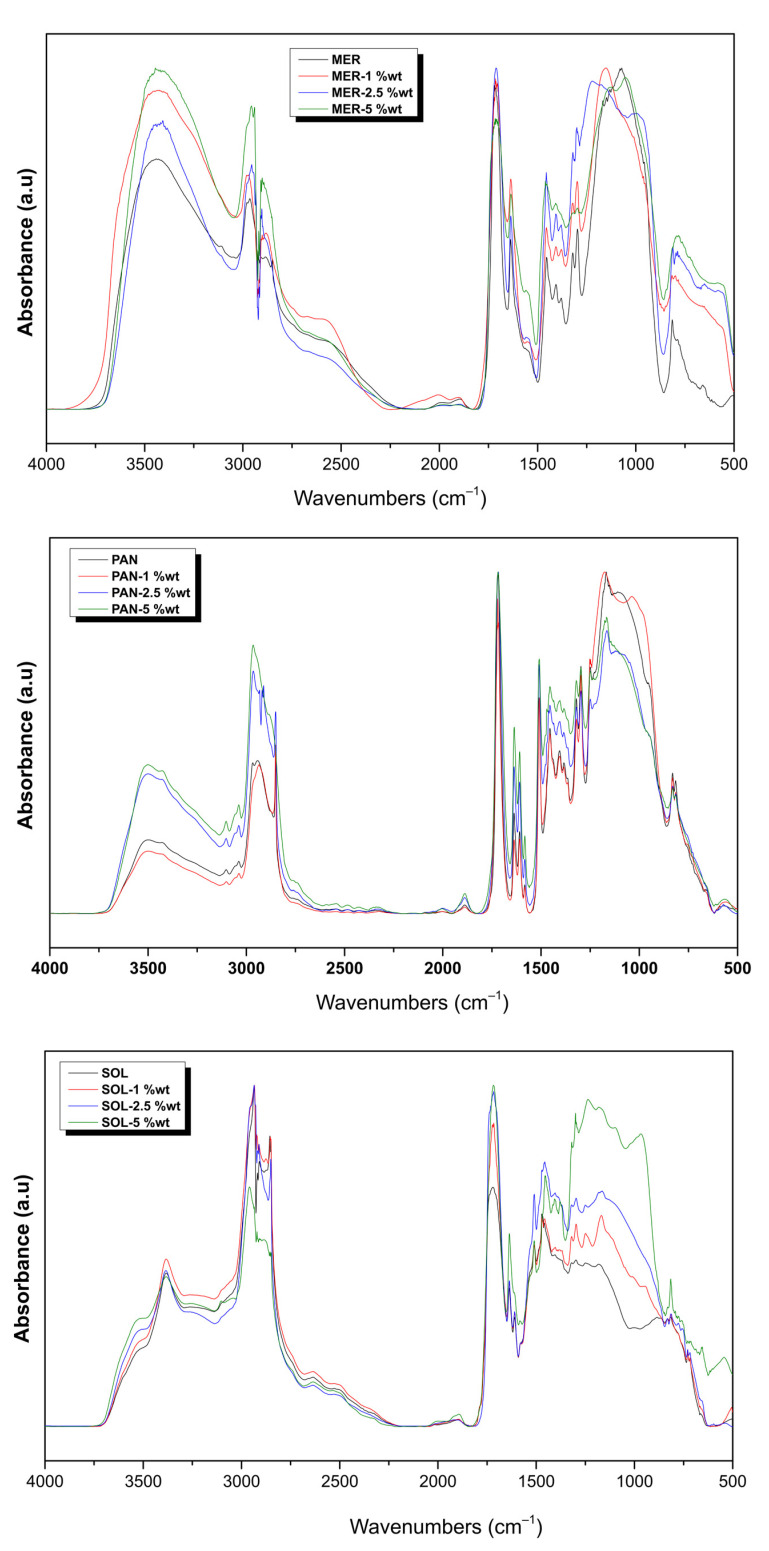
Comparison of the spectra of the three luting cements following the addition of zirconia nanoparticles (ZrO_2_ NPs) at all ratios, right after dual polymerization. MER = Meron plus QM, MER-1wt% = Meron plus QM with 1% wt zirconia NPs, MER−2.5wt% = Meron plus QM with 2.5% wt zirconia NPs, MER−5wt% = Meron plus QM with 5% wt zirconia NPs, PAN = Panavia SA Universal, PAN−1wt% = Panavia SA Universal with 1% wt zirconia NPs, PAN−2.5wt% = Panavia SA Universal with 2.5% wt zirconia NPs, PAN−5wt% = Panavia SA Universal with 5% wt zirconia NPs, SOL = Solocem, SOL−1wt% = Solocem with 1% wt zirconia NPs, SOL−2.5wt% = Solocem with 2.5% wt zirconia NPs, SOL−5wt% = Solocem with 5% wt zirconia NPs.

**Figure 2:**
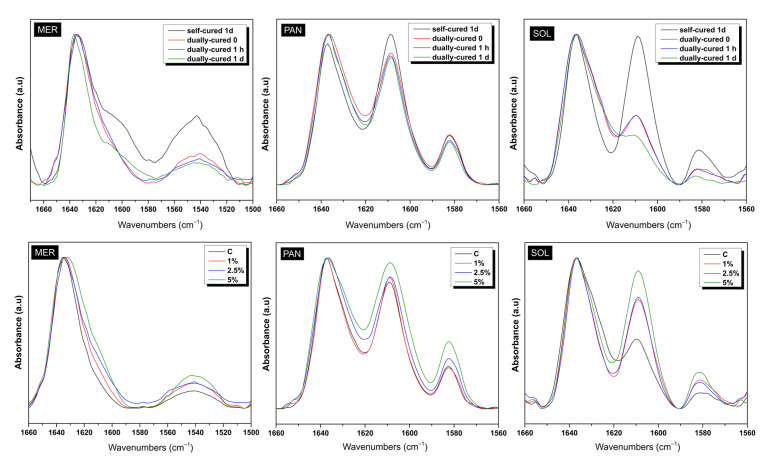
Comparisons of the limited-area spectra for all cements. (**Top**) Effect of curing and (**Bottom**) influence of the nanoparticle (NP) content on the polymerization progress. MER = Meron plus QM, SOL = Solocem, PAN = Panavia SA Universal, C = control without NPs, 1% = 1% wt. NPs, 2% = 2% wt. NPs, 5% = 5% wt. NPs.

Many spectra were collected in order to clarify the influence of each parameter in the progress of polymerization for the materials studied. Figure 1 shows the full spectra recorded for the composite materials right after dual polymerization. The effect of the addition of ZrO_2_ NPs could not be detected in the full spectra, since tetragonal zirconia presents broad bands at about 430–440 cm^−1^ and a weak broad band at around 600–650 cm^−1^ [1,2]. The spectra were not identical in shape or in the intensity of peaks, but it is apparent that all three commercial materials contained similar ingredients in their nature. Parts from the full spectra were isolated to accurately identify each peak. Figure 2 (top) demonstrates the influence of photopolymerization in the MER, PAN, and SOL luting cements. The chart includes the absorptions in the range of 1660–1560 cm^−1^, taken right after mixing (control sample), 1 h and 1 d after self-curing, plus a day after additional photocuring. Figure 2 (bottom), on the other hand, presents the partial spectra of the MER, PAN, and SOL adhesive, dual-cured, when ZrO_2_ particles were added in 1, 2.5, or 5% wt. The values of *DC*% are given by the equation:

$$DC\%=100\cdot \left[1-\frac{{\left(A_{1637/A_{1608}}\right)}_{t}}{{\left(A_{1637/A_{1608}}\right)}_{0}}\right]\;$$

where *A* is the peak area value for the particular peak and time interval, while absorption at 1637 cm^−1^ corresponds to C=C and at 1608 cm^−1^ to C···C bonds. 

The integration of the peaks for quantitative results via FTIR is a demanding task, taking into consideration the bonds found, the shape, intensity, and limits of the peaks. In the present study, the great absorption at 1637 cm^−1^ illustrates the presence of vinyl bonds C=C in dimethacrylates, which react and produce polydimethacrylate networks. The reaction occurs with self-curing and/or with photocuring, given the correct initiators: BPO for self-polymerization and camphorquinone for photopolymerization (Table 1). Apart from the area beneath the 1637 cm^−1^ peak, the simultaneous integration of another neighboring peak, indicating a group that does not participate in the polymerization reaction, is crucial for comparison reasons (internal standard). Thus, the absolute area numbers do not lead to conclusions, unless they are taken as ratios with the areas of another peak of the spectra (elimination of experimental inconsistencies). For PAN and SOL materials, this other peak is the neighboring 1608 cm^−1^, corresponding to aromatic bonds C···C (due to the Bis-GMA monomer, Table 1) and for MER, this is the neighboring 1540 cm^−1^ absorption, corresponding to the N–H bond (due to the UDMA monomer). Table 1 lists the results obtained from the calculations. 

### 2.2. Water Sorption and Solubility

**Figure 3:**
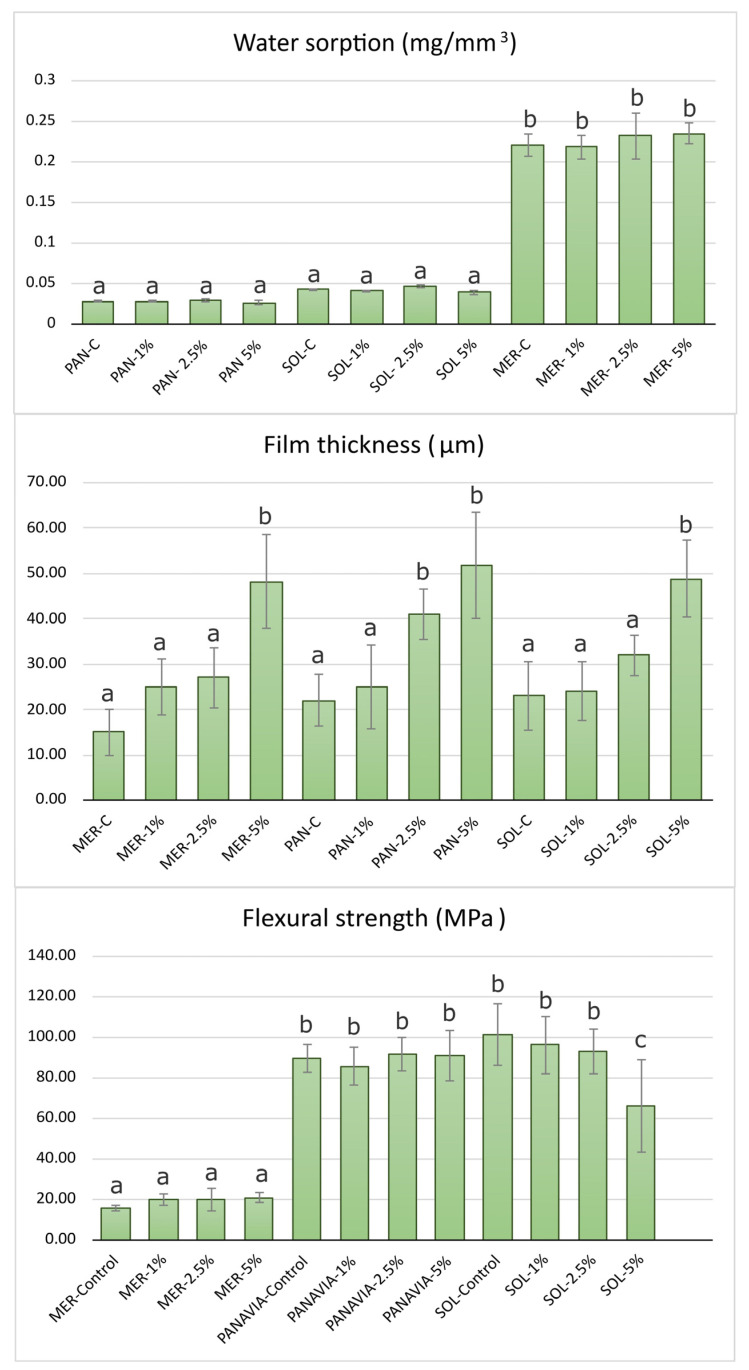
Mean values and SD (error bars) for water sorption, film thickness, and flexural strength of the composite specimens. Different letters suggest significant differences in the mean values. MER = Meron plus QM, MER−1% = Meron plus QM with 1% wt zirconia NPs, MER−2.5% = Meron plus QM with 2.5% wt zirconia NPs, MER−5% = Meron plus QM with 5% wt zirconia NPs, PAN = Panavia SA Universal, PAN−1% = Panavia SA Universal with 1% wt zirconia NPs, PAN−2.5% = Panavia SA Universal with 2.5% wt zirconia NPs, PAN−5% = Panavia SA Universal with 5% wt zirconia NPs, SOL = Solocem, SOL−1% = Solocem with 1% wt zirconia NPs, SOL−2.5% = Solocem with 2.5% wt zirconia NPs, SOL−5% = Solocem with 5% wt zirconia NPs.

As seen in Figure 3, the addition of NPs did not statistically significantly influence the initial *W_sp_* values of all three luting cements. The best performance with the lowest water sorption was measured in the PAN group, followed by the SOL group. The highest values were measured in the MER group (0.22 mg/mm^3^), all within the clinically acceptable levels.

### 2.3. Film Thickness

As seen in Figure 3 the film thickness in all control groups was less than 50 μm and in all cases less than 10 μm above the manufacturer’s measurements. The ranking was MER−C (15 ± 5 μm) < PAN−C (22 ± 5 μm) < SOL−C (24 ± 5 μm) at 0.05. After the addition of nanoparticles at 1% wt, no significant differences were found. The ranking after the addition of 2.5% wt ZrO_2_ NPs was MER−2.5 (25 ± 8 μm) < SOL−2.5 (32 ± 4 μm) < PAN−2.5 (41 ± 5 μm). Finally, the visual observation that the viscosity decreased after the addition of 5% nanoparticles predicted the significantly increased film thickness in all groups (Figure 3).

### 2.4. Estimation of Flexural Strength 

The flexural strength results showed a significant difference between MER and the two other resin cements, PAN and SOL. The addition of 2.5% of nanoparticles resulted in higher values for the MER group (20 ± 5 MPa). The PAN and SOL groups resulted in similar values in almost all groups except for SOL−5%. In all cases, the different % amounts of nanoparticles did not significantly affect the flexural strength except for the SOL group, where a 5% wt addition significantly reduced the strength, while for the MER group, all concentrations led to an increase in the flexural strength but not on a significant level.

### 2.5. Shear Bond Strength Results

A total of 24 groups were subjected to the SBS test. The initial values before thermocycling and the final values for each group are both summarized in Table 2. Groups MER−C, MER−2.5, and SOL resulted in the lowest values of all groups studied. On the other hand, groups SOL-G, SOL−G−2.5, PAN−G, and PAN−G−2.5 exhibited higher values and group PAN−G−2.5 had the highest (22 ± 5 MPa). All other groups ranged between 5 and 10 MPa.

For MER three-way ANOVA showed that the presence of Gluma had a significant effect on the shear bond strength (*p* = 0.006), while this was not the case for the addition of NPs. However, the interaction of the addition of NPs and TC presented a significant effect (*p* = 0.045), and pairwise comparisons demonstrated a significant difference between MER-C and MER−2.5% after thermocycling. Regarding the PAN, TC, and Gluma adhesives, they all presented a significant main effect (*p* < 0.001 and *p* = 0.03, respectively) as well as their interaction (*p* < 0.01). Similarly to MER, the presence of NPs did not affect the SBS. The main effects of Gluma and TC were also significant for SOL (*p* < 0.001) as well as their interaction (*p* = 0.05). The presence of NPs did not affect the shear bond strength mean values in a statistically significant manner.

After thermocycling, there was a significant decrease in SBS in all groups. The lowest values were observed in MER−C−TC, MER−2.5−TC, SOL−C−TC, and SOL−2.5−TC. The highest values were observed in PAN−C−TC, PAN−2.5−TC, and MER−G−C−TC. All the other groups values ranged from 0 to 6.7 MPa. It was observed that TC was detrimental to groups with high initial values except for the case of MER−G−C. Only the PAN−C, PAN−2.5, and MER−G−C groups showed resistance in thermal degradation after 5000 cycles. Three groups with 2.5% nanoparticle addition (MER−G 2.5, SOL−2.5, PAN−G−2.5) showed a significant reduction in SBS, but showed a higher ranking than their control groups after TC.

### 2.6. Failure Mode Results

**Figure 4:**
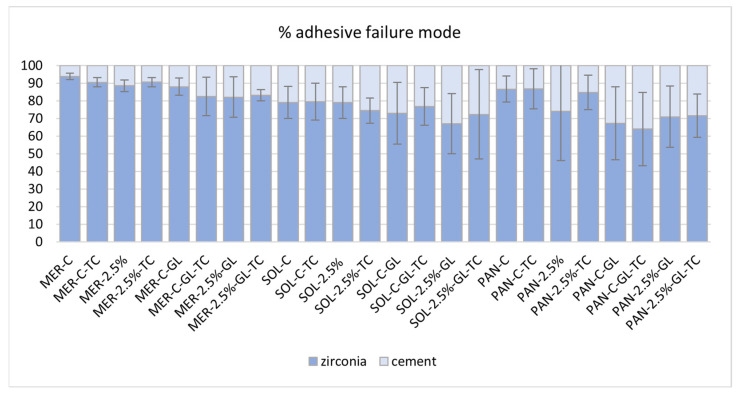
Adhesive failure mode (ADFM %) as calculated from stereoscope images using image analysis software. MER−C = Meron plus QM Control, MER−C−TC = Meron plus QM Control after thermal cycling, MER 2.5% = Meron plus QM with 2.5% zirconia NPs, MER 2.5%−TC = Meron plus QM with 2.5% zirconia NPs after thermal cycling, MER−C−GL = Meron plus QM Control after Gluma application, MER−C−GL−TC = Meron plus QM Control after Gluma application and thermal cycling, MER 2.5%−GL = Meron plus QM with 2.5% zirconia NPs after Gluma application, MER 2.5%−GL−TC = Meron plus QM with 2.5% zirconia NPs after Gluma application and thermal cycling, SOL−C = Solocem Control, SOL−C−TC = Solocem Control after thermal cycling, SOL 2.5% = Solocem with 2.5% zirconia NPs, SOL 2.5%−TC= Solocem with 2.5% zirconia NPs after thermal cycling, SOL−C−GL = Solocem Control after Gluma application, SOL−C−GL−TC = Solocem Control after Gluma application and thermal cycling, SOL 2.5%−GL = Solocem with 2.5% zirconia NPs after Gluma application, SOL 2.5%−GL−TC = Solocem with 2.5% zirconia NPs after Gluma application and thermal cycling, PAN−C = Panavia SA Universal Control, PAN−C−TC = Panavia SA Universal Control after thermal cycling, PAN 2.5% = Panavia SA Universal with 2.5% zirconia NPs, PAN 2.5%−TC = Panavia SA Universal with 2.5% zirconia NPs after thermal cycling, PAN−C−GL = Panavia SA Universal Control after Gluma application, PAN−C−GL−TC = Panavia SA Universal Control after Gluma application and thermal cycling, PAN 2.5%−GL = Panavia SA Universal with 2.5% zirconia NPs after Gluma application, PAN 2.5%−GL−TC = Panavia SA Universal with 2.5% zirconia NPs after Gluma application and thermal cycling.

**Figure 5:**
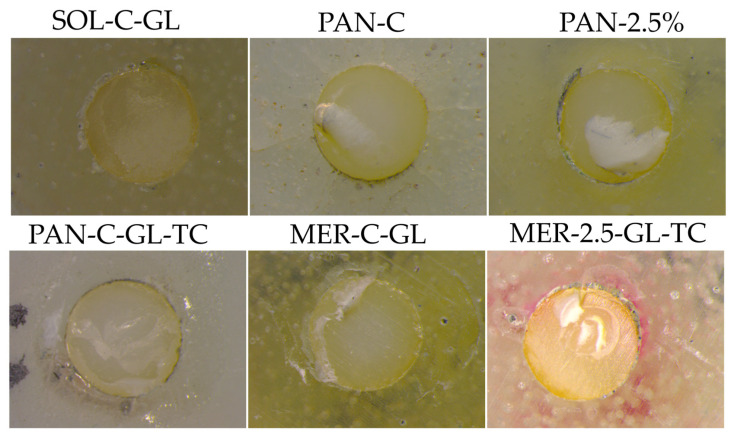
Indicative images from the stereomicroscope of different types of adhesive failure modes. SOL−C−GL = Solocem Control after Gluma application, PAN−C = Panavia SA Universal Control, PAN−2.5% = Panavia SA Universal with 2.5% wt zirconia NPs, PAN−C−GL−TC = Panavia SA Universal Control after Gluma application and thermal cycling, MER−C−GL = Meron plus QM Control after Gluma application, MER−2.5−GL−TC = Meron plus QM WITH 2.5% zirconia NPs after Gluma application and thermal cycling.

Using image analysis, the adhesive failure mode (ADFM%) was calculated from every specimen in each group and is presented in Figure 4. Only PAN groups presented significantly lower ADFM% than the other groups (64–86%). Groups PAN-GL and PAN 2.5%−GL showed the best failure mode results of 67% and 71% respectively. MER groups without adhesive presented the least favorable results (88–94%). In the MER groups, the high ADFM% was decreased after GL treatment (82–83%), except for group MER−GL−TC (88%). In the SOL and PAN groups, GL treatment resulted in a decrease in ADFM. Figure 5 illustrates indicative snapshots of the failed samples through stereomicroscopic imaging. 

## 3. Discussion

According to the results of the present study, the first null hypothesis was partially rejected because several physicochemical properties (film thickness, flexural strength) of the tested luting cements were affected by the addition of NPs. On the other hand, water sorption was not influenced by the addition of 2.5% tetragonal zirconia nanoparticles. Film thickness was influenced in the PAN and MER groups, while SOL showed a non-significant increase. In the flexural strength tests, a significant increase was measured only in the MER groups. The second null hypothesis partially failed to be rejected, since the shear bond strength was not affected by the addition of NPs before TC, but was affected in two of the three cements after TC. Finally, the third null hypothesis was rejected, since almost all groups presented reduced SBS values after TC.

In the present study, the selection of adhesive materials was based on their different compositions and represented a wide range of clinically accepted luting cements used for zirconia substrates. 

Zirconia NPs were fabricated following a sol–gel technique as previously published [38]. In general, YSZ (tetragonal) nanoparticles present chemical inertness, low thermal conductivity, and offer higher mechanical properties than monoclinic zirconia particles [39]. The NPs used in the present study presented low agglomeration and sizes ranging from 20 to 50 nm [38].

After observing Figure 1, one can note the main groups included in all cases: at 3425 cm^−1^, the –OH groups were shown; at the 2960–2870 cm^−1^ region, all the –CH_3_, –CH_2_–, and C–H bonds absorbed; the sharp and intensive peak at 1716 cm^−1^ corresponded to the C=O group; the medium peak of 1637 cm^−1^ indicated the C=C absorption; and the one at 1608 cm^−1^ the aromatic double bonds. At lower wavenumbers, the peak at 1540 cm^−1^ was attributed to N–H present in the urethane dimethacrylate monomer, while the great peak at 1152 cm^−1^ corresponded to the C–O bonds [3,4]. However, the fingerprint region beneath 1200 cm^−1^ was hard to evaluate in detail and the overlapping of various peaks was evident. 

Regarding the presence of the inorganic fillers in the composite materials, the peak at 1298 for the Si–CH_3_ bond was apparent and the Si–O–Si group absorbed in the region 1130–1000 cm^−1^ was difficult to identify alone, while the shoulder at 953 cm^−1^ was attributed to the stretching vibration of this group. The Si–OH group was included in the 3452 cm^−1^ vast peak, while the small peak at 815 cm^−1^ was also characteristic of amorphous silica. 

Particularly for the PAN adhesive, the absorptions located in the area beneath 1200 cm^−1^ also included the P–O vibrations from the –PO_3_^2−^ group. The 10-MDP monomer bears a methacrylate structure on one side and a –PO_3_^2−^ group on the other side. The –OH absorptions are located in the 3400 cm^−1^ area of the PAN spectra. The main P–O absorption was found at 1086 cm^−1^, while at 1249 cm^−1^ and 945 cm^−1^, two slight shoulders could be attributed to the same bond vibration [40]. There were also two apparent small peaks, seen only for the PAN adhesive, at 830–813 cm^−1^, but the literature provides no evidence for P–O absorptions in this area. 

Regarding the presence of ZrO_2_ in the composite materials, the Zr–O bond was found in the regions where the Si–O bond also absorbs. Thus, for the three composite materials already containing silicates, it was hard to isolate the Zr–O bond. The small difference the authors noticed concerned the appearance of a small peak at the right-end of the spectra. In fact, the addition of ZrO_2_ provided a shoulder for the MER adhesive at 553 cm^−1^, for the PAN adhesive at 561 cm^−1^, and for SOL at 541 cm^−1^. The change was obvious mainly in the 5% wt sample of each case. The literature is lacking evidence regarding the IR absorptions of ZrO_2_ in the fingerprint region due to the numerous and noisy recordings, an issue the authors also faced. 

Figure 2 (top) points out the influence of photocuring on the already self-cured composites. The additional energy provided to the films had a great effect, since the area of the peak of C=C shown was minimized relative to the area of the unreactive bonds. For the SOL material, the *DC* of 77% was achieved once the photocuring was fulfilled, while for the PAN and MER cements, it was 64 and 60%, respectively, almost double the *DC*% values achieved after their self-polymerization. Thus, it can be concluded that the intervention of photopolymerization after self-polymerization is beneficial for the hardening of the material (Table 2) [36]. The influence of the 10-MDP monomer for the PAN adhesive cannot be evaluated by the *DC*% results, since the exact organic content of the composites is unknown to the authors (the dimethacrylate percentages compared to 10-MDP). Likewise, the influence of the 4-META monomer on the SOL adhesive cannot be estimated in the *DC*% results, since the exact synthesis of the matrix is unknown [41]. All in all, the influence of light curing is effective for composite materials given the fact that the analogous photo-initiators are present in their composition. 

It is worthy to note that as MER does not include a photo-initiator (usually camphorquinone), photocuring does not result in new radicals for the monomer reactions. Thus, MER may not be considered as a “dual adhesive”, and it may not be appropriate to call it a “dual adhesive”. However, the authors believe that after the application of light onto films, a second curing occurs, since the beam also provides thermal energy, apart from the quanta specific for light-activation. The energy provided activates the “frozen” macro-chains to be mobilized, so new monomers react, and further curing is promoted. 

Figure 2 (bottom) demonstrates the influence of the addition of ZrO_2_ NPs in the cements when mixed at various ratios. The calculation of the *DC*% values proves that the addition of 1% wt ZrO_2_ NPs does not influence the degree of polymerization, either after self-curing only or dual-curing. However, the addition of 2.5 or 5% wt of zirconia powder does affects the polymerization progress by obstructing the conversion of C=C to C–C, especially for the PAN composites. The values obtained for all materials when 2.5 or 5% wt zirconia was added were diminished to a lower conversion by 10–15% (Table 1). Thus, regarding the polymerization efficiency, the lowest addition tested is recommended, taking into consideration that the mixing occurred manually, and that mechanical mixing might be more effective. The reasons why a filler addition might prevent the evolution of the radical reactions are two: (a) a possible blocking of irradiation into the mass of the material reinforced with extra NPs, preventing light from finding the initiators to react, and, (b) the oligomers that have been produced do not move easily into the stiffer mass of the composite in order to attack the monomers and attach them to the macro-chains. Consequently, the ZrO_2_-reinforced composites present lower *DC*% values compared to the untreated materials. 

The integration of pure nanoparticles to commercial products that have already been reinforced by a high load of silica fillers seems promising. The concept of this research was based on the possible direct bond of zirconia to phosphoric or carboxylic adhesive monomers [32,41]. In addition, zirconia nanoparticles enhance radiopacity [42,43] and transform luting agents to more viscous materials, and probably increase the micro-hardness [44]. Although silanization has been proposed for inorganic fillers to increase the mechanical properties of composite resins [45], because silica particles are hydrophobic, zirconia NPs are hydrophilic and can be used without further silanization [46]. However, the silanization of zirconia nanoparticles has recently been proposed in the reinforcement of PMMA and adhesives with promising results [23,28]. Moreover, in our study, if those particles were silanized, the advantage of direct contact with the MDP or 4-META adhesive monomer could be lost. In addition, the influence of zirconia nanoparticles on the color stability of cements should be carefully considered, as contemporary zirconia materials show high translucency that could affect the final shade of the cemented restoration [47].

The initial values of film thickness in all luting agents were far lower than the ISO requirements (<50 μm). The lowest value was measured in the MER−C group (15 μm). The addition of nanoparticles increased the film thickness, but it was found to be statistically significant only at the 5% concentration for all cements and 2.5% for PAN. The results of this study are comparable to other recent or earlier studies since the film thickness of RMGIs range from 15 to 50 μm [20,48,49,50,51]. The particle size of inorganic fillers determines the limits of the minimum film thickness. Although the SOL group contained a smaller filler size than PAN (5 μm vs. 20 μm), our measurements revealed a similar film thickness in these groups.

The increase in the film thickness could be partially explained by the extended mixing time of the luting agents to homogenize the nanoparticles in the mass of all groups. The visible increase in viscosity during mixing could also explain the increased values in film thickness after NP insertion, which could be attributed to NP agglomerations possibly introduced during hand-mixing. However, with a 1 and 2.5% addition in all groups, this resulted in a clinically acceptable film thickness. In the PAN group, a significant increase was measured in the PAN−2.5 and PAN−5 groups. The initial film thickness agreed with all of the manufacturers’ internal studies. In the SOL group, only 5% of nanoparticles showed a significant increase in the film thickness. Group SOL−2.5 presented a lower thickness than PAN−2.5, which can partially be explained by the smaller medium size in inorganic filler content.

The analysis of the water sorption results revealed that resin-based luting agents were far less sensitive to water uptake than the RMGI product. The sensitivity of RMGI to high water uptake has been observed in older generations of both RMGI lining and cementing products [16]. The RMGI luting agent tested in this study exhibited statistically significant higher water sorption in relation to the composite cements, which agrees with most similar studies [52,53]. The addition of 1, 2.5, and 5% NPs resulted in a non-significant increased water sorption for the case of MER. The self-adhesive resin luting agent (PAN) presented the lowest water sorption among all groups, in agreement with the manufacturer. Solocem measurements also confirmed the manufacturers’ claim. Measurements were higher than the PAN group, but not statistically significant. One possible explanation could be attributed to the different monomer composition, since materials that have more HEMA in their composition may have a higher water sorption [54]. The addition of NPs in both the PAN and SOL groups did not cause any statistically significant changes in *W_sp_*.

The initial values of the flexural strength test confirmed the theory that resin based products present higher values than other conventional luting agents or even resin-modified glass ionomers [55]. The group SOL presented the highest FS values and confirmed the internal studies of the manufacturer (110 MPa). However, the addition of 5% nanoparticles significantly reduced FS, while all other concentrations had almost no effect in the *σ* values. In all groups reinforced with NPs, it should be noted that hand mixing is a technically sensitive method influencing some mechanical properties compared to automix protocols [48]. In the PAN group, the manufacturer’s claim was also confirmed (90 MPa) and NP addition did not affect the values. Our measurements agree with a recent study that demonstrated values of 87.8 MPa in the self-cure mode and 100.7 MPa in the dual-cure mode of preparation [56]. The MER group showed low values in the control samples, below the ISO 9917-2:2017 acceptance values (20 MPa), but all groups showed higher levels of FS after NP addition (20 MPa), although this was not statistically significant. A recent study demonstrated that MER presents higher FS (37.5 MPa) and this value was further optimized after TC to 43.8 MPa [57]. The significant difference compared to our results could be attributed to differences in the experimental setup. It is also known that in RMGIs, acid–base and polymerization reactions are antagonistic [57]. 

As mentioned in the Materials and Methods (Section 4), zirconia specimens designed for the SBS test were not polished to simulate clinically relevant conditions. The internal surface of a zirconia crown cannot be efficiently polished clinically. However, most research papers have preferred to polish the zirconia surface to standardize the initial roughness of the specimens [11,58,59].

The use of primer (GLUMA adhesive) in all groups resulted in higher SBS. In the clinical application of self-adhesive luting agents, most manufacturers do not recommend additional adhesive primers. However, recently, manufacturers have suggested a 10-MDP containing primer to promote adhesion to substrates such as zirconia [60,61].

The lowest values were recorded in the MER group, and many spontaneous detachments before the SBS test were observed. However, this measurement (~4 MPa) was higher than that of the adhesive-free resin cement shown in previous studies [62], and implies a weak chemical affinity to zirconia substrates. RMGI cements incorporate poly-carboxylate groups that might adhere to zirconia surfaces [35]. Fracture analysis showed an almost exclusively adhesive failure mode (ADFM 82–90%). However, in some samples, the remnants of RMGI were visible under 60X magnification (Figure 5). The universal adhesive significantly increased SBS, which was performed against the recommendations of the manufacturers. The unexpected resistance to TC denotes that the 10-MDP and 4-META adhesives were both active to the zirconia surface and glass ionomers. The increased values of MER-GL after TC imply a post-polymerization effect of the cement or a resistance to water absorption due to the high initial water sorption of MER. The addition of NPs did not influence the initial values of SBS in any of the MER groups. Moreover, the reinforced group (2.5% NP) was more sensitive to TC, minimizing the beneficial effect of Gluma. A possible delay in the light curing due to NP insertion and extended mixing time can partially explain these results. Additionally, the setting of RMGIs is more complex since acid–base and polymerization reactions are antagonistic [63]. Since the RMGI luting agents are free of adhesive monomers, weak bonds between carboxylate groups and NPs can occur. However, a possible reaction of NPs can be expected in the interface since the primer contains MDP and 4-META monomers. Moreover, the observation that the RMGI was more viscous after the NP load could also partially explain the reduced SBS.

The initial measured SBS values of SOL were similar to MER. The addition of NPs increased the SBS values but after TC, the SBS dropped detrimentally in both groups. A significant increase in SBS after the application of the primer was observed (+20 MPa). It seems that the manufacturers’ recommendation for an additional primer for adhesive cementation (one coat 7.0/Coltene-MDP and nanofillers) was confirmed by our results. On the other hand, after TC, a significant negative influence in SBS was also noticed. A possible incompatibility or susceptibility of this material combination to water intake could explain this effect. The reinforced with NP sample groups after TC presented similar SBS values to the non-adhesive group.

The PAN group presented the highest values of all groups. Without any additional treatment, the initial values were maintained after TC, showing an exceptional resistance to thermal stress and hydrolysis. It was demonstrated from the *W_sp_* results that the material is extremely stable with a minimum water sorption. Moreover, this product is the only self-adhesive luting agent that incorporates two adhesives, one containing 10-MDP and one novel adhesive with a long carbon chain silane (LCSi) [61]. The addition of a primer was beneficial, resulting in higher initial SBS values. However, after TC, a totally different behavior was revealed. The high values dropped by 77% in the PAN−G group after TC indicated a vulnerable combination or an unexpected incompatibility of these two products. The manufacturer recommends another additional primer for zirconia bonding that increases the SBS to zirconia. Moreover, the zirconia surface contains no silica, neutralizing the possible benefits from LCSi, if the surface is treated with a silicatization sandblasting technique, as proven in an earlier study [62]. The addition of NPs presented a beneficial initial effect in this group, but after TC, a detrimental effect in SBS was observed. In this group, the presence of MDP in both the primer and luting agent can partially explain the high initial values in SBS. This observation may be attributed to the increased complexity or possible incompatibility with 4-META and LCSi monomers in the primer and luting agent, respectively. The interaction of NPs with 10-MDP in both the luting agent and primer could partially explain this finding. Further studies in a chewing simulator to evaluate resistance to cyclic fatigue should be performed to confirm these findings [64]. 

Only three out of 12 groups showed resistance to TC. The water sorption and thermal stresses caused a detrimental decrease in the SBS values. In the MER group, the primer treatment presented a significant resistance in TC. In the SOL group, a minor effect was observed, while the combination of the primer did not withstand the effect of TC. PAN was resistant to TC, and the product after the addition of NPs showed a significant decrease after TC. The combination of PAN with Gluma presented a susceptibility to water and TC. It seems that incompatibility or extreme hydrophilic behavior could explain this finding and that the addition of NPs intensifies the influence of water in this group. 

## 4. Materials and Methods

### 4.1. Preparation and Characterization of Nanoparticles

Yttria-stabilized zirconia nanoparticles (ZrO_2_-7% wt Y_2_O_3_) were synthesized by the sol–gel method using zirconium oxychloride octahydrate (ZrOCl_2_·8H_2_O) and yttrium nitrate hexahydrate (Y(NO_3_)_3_·6H_2_O) as reagents. Raw materials were dissolved in distilled water, and then ethylene glycol (brand) and an aqueous citric acid concentrate (brand) were added under heating and stirring. The molar ratios of citric acid:metal (Zr) was 3.65 and citric acid:ethylene glycol was 1, respectively. The material was gradually heated to a temperature of 300 °C to eliminate organic materials [65] and then sintered at 1000 °C for 2 h. To avoid agglomeration, the obtained NPs underwent ultrasonic treatment in ethanol (brand) for 20 min before application. The characterization of the NPs is presented elsewhere [38].

### 4.2. Zirconia Specimen Preparation

Zirconia specimens were fabricated using CAD/CAM technology from translucent zirconia blocks (priti^®^multidisc ZrO₂, monochrome, pritidenta^®^ GmbH, Leinfelden-Echterdingen, Germany, 5Y-TZP) following the manufacturer’s instructions for sintering. The final dimensions of the zirconia specimens were 4 mm in diameter and 6 mm in height. The zirconia specimens designed for the shear bond strength (SBS) test were not polished (to simulate clinically relevant conditions) or sandblasted and were embedded in a transparent acrylic resin (Jet Liquid, Lang) using Plexiglas molds of 16 mm. The specimens were ultrasonicated in isopropyl alcohol (brand) for 15 min and finally dried with oil-free air 0.25 MPa for 10 s.

### 4.3. Incorporation of Zirconia NPs into Luting Cements 

One resin-modified glass ionomer and two different types of composite luting cements were used: (a) RMGI cement (Meron plus QM, VOCO, Cuxhaven, Germany), (b) self-adhesive composite luting cement containing 4-META adhesive monomers (Solocem, Coltene, Altstätten, Switzerland), and (c) self-adhesive composite luting cement containing the adhesive monomer 10-MDP (Panavia SA Universal, Kuraray, Japan) (Table 3). An additional Universal primer Gluma (Kulzer, Germany) was applied for the shear bond strength test.

**Figure 6:**
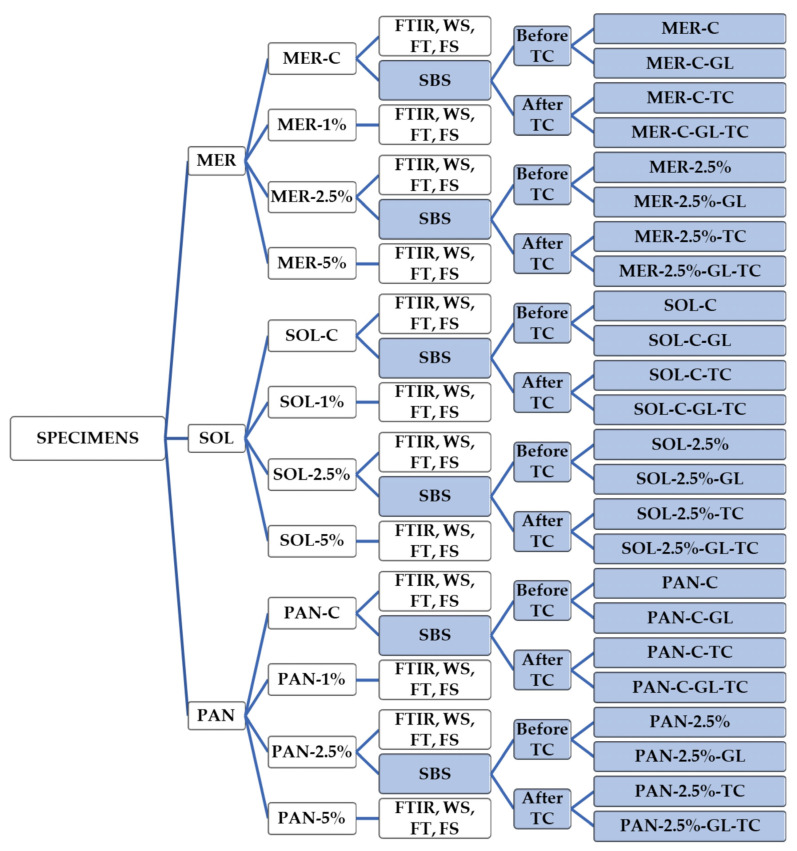
Schematic representation of the study groups and performed analysis. MER = Meron QM, SOL = Solocem, PAN = Panavia SA Universal, GL = Gluma, C = Control, FTIR = Fourier Transform Infrared Spectroscopy, WS = water sorption, FT = film thickness, FS = flexural strength, SBS = shear bond strength test, TC = thermocycling.

Zirconia NPs were added in percentages of 1, 2.5, and 5% wt. The incorporation of the powder zirconia NPs in several ratios was performed after appropriate weighing (Mettler Toledo A250 balance, ±0.0001 g) and mixed manually with the two pastes simultaneously. After initial self-curing, the materials were then additionally photopolymerized. A curing photopolymerizing device with a light intensity of 1200 mW/cm^2^ at the spectral range of 380–515 nm was used (Curing Pen-E, Eighteenth, Changzhou Sifary Medical Technology Co. Ltd., Changzhou, China) [38]. Different specimens were prepared depending on the test as described in the following paragraphs and shown schematically in Figure 6.

### 4.4. Investigation of Physical and Mechanical Properties of Modified Luting Cements

#### 4.4.1. FTIR Analysis

To evaluate the effects of the addition of NPs on the photopolymerization of the materials, FTIR analysis was performed on a Spectrum One (Perkin Elmer, Waltham, MA, USA) instrument. First, the spectrum of the pastes of each composite cement was recorded. Then, the mixing of the two pastes of each composite occurred, as indicated by the manufacturers, and the polymerized material produced was analyzed (self-cured materials). Self-cured materials were analyzed immediately (time = 0), after 1 h (time = 1 h), and after 24 h (time = 24 h). In a second step, the materials were evaluated after dual polymerization (5 × 10 s photopolymerization). For all cases, right after mixing, the pastes were pressed between two glass plates (5 mm thick) covered with commercial polyethylene sheets and a thin film was shaped to be analyzed. The cured film was placed between two round NaCl IR crystals (Sigma-Aldrich, Lot #: z123595-1EA, Batch #: 3110, 25 mm × 4 mm) for the transmittance recordings. The spectral range was 4000–600 cm^−1^, the resolution at 4 cm^−1^, after 32 scans. No external natural light was available during the experiments and the samples were placed in a dry and shady place until 24 h after polymerization.

#### 4.4.2. Evaluation of Water Sorption and Solubility

Five disk specimens (15 mm ×1 mm) of each group were fabricated and photopolymerized by overlapping irradiation at eight points according to manufacturer’s instructions. The specimens were placed in a desiccator for 24 h and their initial weight and volume was recorded. Then, the specimens were immersed in distilled water for 7 d at 37 °C and weighed with an accuracy of 0.001 mg. The values of water sorption (*W_sp_*, mg/mm^3^) were calculated using the following equations (ISO/TS 16506:2017):

$$W_{sp}=\frac{m_{i}-m_{f}}{V}$$

where *m_i_* is the specimen mass before immersion (mg); *m_f_* is the specimen mass after immersion (mg); and *V* is the specimen volume before immersion (mm^3^).

#### 4.4.3. Estimation of Film Thickness

To calculate the film thickness, an appropriate amount of each cement (0.05 mL), with or without the addition of NPs, was placed between two optically flat square glass plates, each having a contact surface area of 225 mm^2^ and a uniform thickness of 5 mm. A customized loading device with 150 N force was manufactured to fulfill the ISO/TS 16506:2017 and ISO 9917-2:2017 requirements. Specimens were photopolymerized at the center of the upper glass plate for twice the exposure time, as recommended by the manufacturer. The combined thickness of the two glass plates with (T1) and without the cement (T2) film was measured using a micrometer with accuracy of 0.5 μm, while the film thickness was estimated as the difference between these two values.

#### 4.4.4. Determination of Flexural Strength

**Figure 7:**
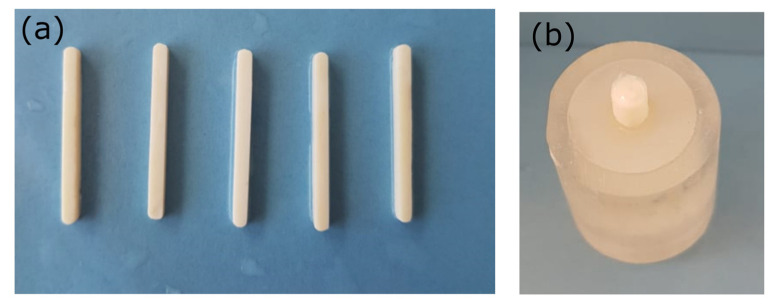
(**a**) Specimens prepared for flexural strength, (**b**) embedded zirconia specimens after cementation for shear bond strength test.

Ten bar-shaped specimens of 25 mm length, 2 mm thickness, and 2 mm width (Figure 7a) of each group of materials were fabricated using a Teflon mold covered with glass plates before polymerization (Figure 7a). These underwent a 3-point bending test with a constant crosshead speed 0.5 mm/min and rate of loading of 50 N/min on a Instron 3344 dynamometer. Flexural strength (MPa) was calculated according to the equation (ISO/TS 16506: 2017):

$$\sigma =\frac{3FL}{2bh^{2}}$$

where *F* is the load until failure in N; *L* is bar length; *b* is width; and *h* the height in m. Failed specimens were observed by SEM.

#### 4.4.5. Preparation of Specimens for Adhesive Bonding

All zirconia surfaces were cleaned using ethanol and thoroughly dried, and a thin layer of the universal primer (Gluma, Kulzer) containing both the 10-MDP and 4-META adhesive monomers was applied in half of the specimens and photopolymerized. Then, each cement was mixed according to the manufacturer’s instructions, while all NP reinforced cements were hand mixed. An optimal NP content of 2.5 wt% was chosen, based on the results derived from the previous experiments. The cement was placed in a split mold to standardize a 3 mm diameter adhesive area (Figure 7b). Each cement was photo-polymerized according to the manufacturer’s instructions as described in Section 4.3. The mold was carefully removed, and each specimen was held in a humid environment (100% distilled water) at 37 °C to maintain the standard temperature. Half of these specimens underwent additional thermal aging, following 5000 cycles between 5 and 55 °C with a dwelling time of 20 s and a rest time of 10 s.

#### 4.4.6. Shear Bond Strength to Translucent Zirconia Substrate

All bonded specimens were subjected to the shear bond strength test using a Universal testing machine (Instron 3344; Instron, Burlington, ONT, Canada) with a cross-head speed of 1 mm/min until failure. Specimens were assembled in a custom-made device according to ISO 29022 and the Ultra-tester (Ultradent Products, Inc., South Jordan, UT, USA). The maximum shear force was measured for each specimen and the stress was calculated following the equation *σ* = F/A, where F is the force applied and A is the cross-sectional area of the specimen. 

#### 4.4.7. Failure Mode Analysis

All specimens were observed under a stereomicroscope (M80, Leica, Weltzar, Germany) at 25× magnification to evaluate the failure mode. Failure mode was quantified as the percentage % of the cement-free intact zirconia surface relative to the total zirconia bonded area corresponding to the adhesive failure mode (ADFM%) by image analysis utilizing Photoshop CC software (Adobe Systems, San Jose, CA, USA).

### 4.5. Statistical Analysis

The values reported in the tables and figures represent the mean values ± standard deviation of the replicates. To determine the effect of NP addition on water sorption, film thickness, and flexural strength, one-way analysis of variance (ANOVA) was applied, followed by the Bonferroni test for multiple comparisons (significance level set at a = 0.05). Normal distribution was verified with the Shapiro–Wilks test and the equality of variances with Levene’s test. To test the effect of the addition of NPs, adhesive (Gluma), and thermocycling on the bond strength to zirconia, three-way ANOVA was used with pairwise comparisons (significance level set at a = 0.05).

## 5. Conclusions

Within the limitations of this in vitro study, the following conclusions were drawn:

1. The addition of NPs did not significantly change the physicochemical and mechanical properties of the investigated luting cements, except for the case of the RMGI cement, where a significant increase in flexural strength was recorded.
2. The addition of NPs at the concentration of 2.5% wt increased the film thickness in all luting agents, however, the values were kept below 30 μm for the RMGI, 40 μm for 10-MDP, and 35 μm for the 4-META cement.
3. The application of 1% wt NPs did not significantly affect the *DC*% values for all of the composite cements, but greater amounts resulted in a dose dependent reduction in the *DC*% values up to 7.2% for the 4-META and 15.5% for 10-MDP cement.
4. The application of an adhesive primer increased the initial SBS values significantly for all commercial products, however, it was beneficial only in RMGI after thermocycling (~16.12% increase).
5. Thermocycling presented a detrimental effect on most of the groups after the addition of NPs.
6. The 10-MDP-containing luting cements demonstrated higher SBS values compared to the RMGI cements and luting cements with 4-META.

## Figures and Tables

**Table 1:** The *DC*% results derived from the FTIR calculations for all materials when ZrO_2_ powder was added in the mixture (mean ± s.d., n = 2). MER = Meron plus QM, PAN = Panavia SA Universal, SOL = Solocem.

Time	Sample	*DC*%	Sample	*DC*%	Sample	*DC*%
t = 0	MER-C	0.0	PAN-C	0.0	SOL-C	0.0
t = 1 h		30.6 ± 1.2 ^a^		24.6 ± 3.2 ^a^		31.2 ± 0.8 ^a^
t = 1 d		39.9 ± 2.4 ^b^		32.0 ± 2.4 ^b^		41.1 ± 1.1 ^b^
t = 0	MER_dual_	60.6 ± 1.5 ^c^	PAN_dual_	64.4 ± 1.1 ^c^	SOL_dual_	77.8 ± 2.6 ^c^
t = 1 h		63.9 ± 0.8 ^c^		63.5 ± 0.8 ^c^		78.8 ± 2.4 ^c^
t = 1 d		62.6 ± 1.2 ^c^		71.8 ± 1.5 ^d^		78.5 ± 2.4 ^c^
t = 0	MER-1_dual_	58.3 ± 2.0 ^c,d^	PAN-1_dual_	57.4 ± 2.3 ^e^	SOL-1_dual_	75.5 ± 1.2 ^c^
t = 1 h		58.6 ± 1.0 ^d^		60.2 ± 2.1 ^c,e^		76.2 ± 1.6 ^c^
t = 1 d		59.4 ± 1.6 ^c,d^		61.3 ± 1.8 ^c^		77.0 ± 0.9 ^c^
t = 0	MER-2.5_dual_	56.8 ± 2.5 ^d^	PAN-2.5_dual_	53.3 ± 3.1 ^e^	SOL-2.5_dual_	66.3 ± 2.1 ^e^
t = 1 h		59.3 ± 0.6 ^c,d^		55.1 ± 2.9 ^e^		68.4 ± 0.8 ^e^
t = 1 d		60.6 ± 0.7 ^c,d^		56.3 ± 2.4 ^e^		73.8 ± 1.9 ^c,e^
t = 0	MER-5_dual_	50.8 ± 2.4 ^e^	PAN-5_dual_	55.1 ± 2.5 ^e^	SOL-5_dual_	61.7 ± 3.8 ^e^
t = 1 h		54.4 ± 1.9 ^e^		57.3 ± 1.0 ^e^		66.6 ± 2.1 ^e^
t = 1 d		59.4 ± 1.9 ^c,d^		58.1 ± 0.9 ^e^		71.3 ± 2.6 ^e^

Common lowercase letter as superscript in the same column indicates no significant difference at the *p* < 0.05 level. MER_dual_ = Meron plus QM after dual curing, MER-1_dual_ = Meron plus QM with 1% zirconia NPs after dual curing, MER-2.5_dual_ = Meron plus QM with 2.5% zirconia NPs after dual curing, MER-5_dual_ = Meron plus QM with 5% zirconia NPs after dual curing, SOL_dual_ = Solocem after dual curing, SOL-1_dual_ = Solocem with 1% zirconia NPs after dual curing, SOL-2.5_dual_ = Solocem with 2.5% zirconia NPs after dual curing, SOL-5_dual_ = Solocem with 5% zirconia NPs after dual curing, PAN_dual_ = Panavia SA Universal after dual curing, PAN-1_dual_ = Panavia SA Universal with 1% zirconia NPs after dual curing, PAN-2.5_dual_ = Panavia SA Universal with 2.5% zirconia NPs after dual curing, PAN-5_dual_ = Panavia SA Universal with 5% zirconia NPs after dual curing.

**Table 2:** Results of the shear bond strength tests for all composite cements studied.

	Before TC	After TC		
Sample	SBS (MPa)	SBS (MPa)	Change %	*p* Value
MER−C	3.73 ± 0.40 ^a^	0 ± 0	-	-
MER−2.5	4.01 ± 0.30 ^a^	0 ± 0	-	-
MER−GL−C	13.02 ± 2.98 ^b^	15.12 ± 4.81 ^c^	16.12	0.401
MER−GL−2.5	13.22 ± 3.42 ^b^	6.69 ± 1.74 ^b^	−49.36	0.051
SOL−C	4.69 ± 1.91 ^d^	1.46 ± 0.24 ^f^	−68.91	0.304
SOL−2.5	7.83 ± 4.32 ^d^	0 ± 0	−100	-
SOL−GL−C	20.38 ± 5.63 ^e^	6.45 ± 2.39 ^f^	−68.35	<0.01
SOL−GL−2.5	23.15 ± 1.97 ^e^	4.87 ± 1.38 ^f^	−78.95	<0.01
PAN−C	13.62 ± 5.08 ^g^	12.87 ± 4.41 ^i^	−5.50	<0.01
PAN−2.5	12.78 ± 0.83 ^g^	9.00 ± 5.09 ^i^	−29.59	<0.01
PAN−GL−C	24.31 ± 5.65 ^h^	3.35 ± 1.09 ^j^	−86.20	<0.01
PAN−GL−2.5	29.96 ± 7.74 ^h^	6.03 ± 3.22 ^i^	−79.89	<0.01

Different letters within each column show statistically significant differences among groups of the same cement brand with or without NPs and Gluma (GL) adhesive, while *p* value shows difference before and after TC. MER−C = Meron plus QM, MER−2.5 = Meron plus QM with 2.5% wt zirconia NPs, MER−GL−C = Meron plus QM after Gluma application, MER−GL−2.5 = Meron plus QM with 2.5% wt zirconia NPs after Gluma application, SOL = Solocem, SOL−2.5 = Solocem with 2.5% wt zirconia NPs, SOL−GL−C = Solocem after Gluma application, SOL−GL−2.5 = Solocem with 2.5% wt zirconia NPs after Gluma application, PAN = Panavia SA Universal, PAN−2.5 = Panavia SA Universal with 2.5% wt zirconia NPs, PAN−GL−C = Panavia SA Universal after Gluma application, PAN−GL−2.5 = Panavia SA Universal with 2.5% wt zirconia NPs after Gluma application.

**Table 3:** Composition of the used commercial materials.

Product’s Name	Type of Material	Composition	Filler
Solocem (Coltene, Altstätten, Switzerland)	Self-adhesive, dual-curing composite-based luting cement	Zinc oxide dental glass, urethane-dimethacrylate (UDMA), triethyleneglycol dimethacrylate (TEGDMA), 4-methacryloxyethyl trimellitate anhydride (4-META), 2-hydroxyethylmethacrylate (HEMA), dibenzoylperoxide, benzoylperoxide	Average particle size diameter 2 μm Filler particle size distribution 0.1–5 μm Filling ratio by weight wt% = 69% Inorganic fillers (barium glass, ytterbium trifluoride, spheroid mixed oxide, titanium oxide.)
Meron Plus QM (VOCO, Cuxhaven, Germany)	Self-curing fluoride releasing resin modified glass ionomer cement	Polyacrylic acid peroxide, BHT, methacrylates (hydroxypropyl methacrylate 10–25%, dimethacrylate 5–10%, UDMA 2.5–5%), glycerine	Fluoroaluminosilicate glass 50–100%
Panavia SA Cement Universal (Kuraray, Japan)	Dual-curing fluoride releasing, self-adhesive resin cement	Paste A—10-Methacryloyloxydecyl dihydrogen phosphate (MDP)—Bisphenol A diglycidylmethacrylate (Bis-GMA)—TEGDMA—Hydrophobic aromatic dimethacrylate—2-Hydroxymethacrylate (HEMA)—Silanated barium glass filler—Silanated colloidal silica—dl-Camphorquinone—Peroxide—Catalysts—Pigments Paste B—Hydrophobic aromatic dimethacrylate—Silane coupling agent—Silanated barium glass filler—Aluminum oxide filler—Surface treated sodium fluoride (Less than 1%)—dl-Camphorquinone—Accelerators—Pigments	Inorganic filler (silanated barium glass, aluminum oxide, colloidal silica) is approx. 43 vol%. The particle size 0.02–20 μm
Gluma bond universal (Kulzer, Germany)	Light-curing, self-conditioning all-in-one adhesive	4-META and MDP monomers Methacrylates, Acetone Water	Contains fillers

## Data Availability

Data are contained within the article.
